# Molecular studies of achondroplasia

**DOI:** 10.4103/0019-5413.50856

**Published:** 2009

**Authors:** Risha Nahar, Renu Saxena, Sudha Kohli, Ratna Puri, Ishwar Chandra Verma

**Affiliations:** Department of Genetic Medicine, Sir Ganga Ram Hospital, New Delhi – 110 060, India

**Keywords:** Achondroplasia, FGFR3, molecular study, skeletal dysplasia

## Abstract

**Background:**

Achondroplasia (ACH) is the most frequent form of short-limbed dwarfism, caused by mutations in the FGFR3 gene. It follows an autosomal dominant inheritance, though most cases are sporadic. The molecular techniques are the only available methods to confirm the diagnosis of a skeletal dysplasia. Clinical and radiological features are only suggestive and not confirmatory. The present study was conducted to find out how often the clinical diagnosis of achondroplasia is verified on molecular studies.

**Materials and Methods:**

From 1998 through 2007, we carried out molecular analysis for the two common mutations in the FGFR3 gene in 130 cases clinically suspected to have ACH.

**Results:**

A diagnostic mutation was identified in 53 (40.8%) cases. The common mutation (1138G>A) was present in 50 (94.7%) of the positive cases, while the rare 1138 G>C substitution was found in three (5.3%).

**Conclusion:**

This study shows that confirmation of clinical diagnosis of ACH by molecular genetic testing is essential to distinguish it from other skeletal dysplasias, to plan therapeutic options, and to offer genetic counseling. Management (medical and surgical) in patients confirmed to have ACH, is briefly discussed.

## INTRODUCTION

Achondroplasia (ACH), the most common cause of short-limbed dwarfism in humans, has an estimated birth prevalence of 1 in 26,000–28,000.^1^,^2^ Majority of the cases (80–90%) occur de novo, and these fertile, heterozygous individuals may pass the condition on to their offspring as a fully penetrant autosomal dominant trait. Francomano (1995)^3^ and Wilkin *et al.* (1998)^4^ observed an increased paternal age at the time of conception of the affected individuals, suggesting that the de novo mutations are of paternal origin. Heterozygous ACH is characterized by short limbs, macrocephaly, depressed nasal bridge, frontal bossing, and trident-shaped hands. Radiographs show short tubular bones with squared off iliac wings and short, narrow sciatic notches. The spinal features are short and flat vertebral bodies with distal reduction of the interpedicular distance in the lumbar spine.^5^

The gene responsible for ACH was mapped to chromosome 4 (4p16.3).^5^ This fibroblast growth factor receptor 3 (FGFR3) gene consists of 19 exons and 18 introns. The FGFR3, when mutated, as in ACH, is hyperactivated, resulting in the inhibition of growth of cartilage cells and disturbances in bone growth. Two mutations are commonly observed in the FGFR3 gene.^6^ Both mutations (G>A; G>C) occur at nucleotide 1138 of the cDNA sequence and result in a glycine to arginine substitution at codon 380 (G380R). The present study was conducted to find out the percentage of cases in which the clinical diagnosis of achondroplasia is confirmed by molecular studies.

## MATERIALS AND METHODS

From 1998 through 2007, we received 130 blood samples for mutation analysis of ACH. These cases were referred to us by orthopedic surgeons, geneticists, and pediatricians from all parts of India, as they suspected ACH because of clinical and radiological features such as rhizomelic shortening of the long bones, trident hands, normal trunk length, and contracted base of skull. The subjects were of different age groups ranging from infants to 39 years. Of these, 76 (58.5%) were females and 54 (41.5%) were males.

**Figure 1:**
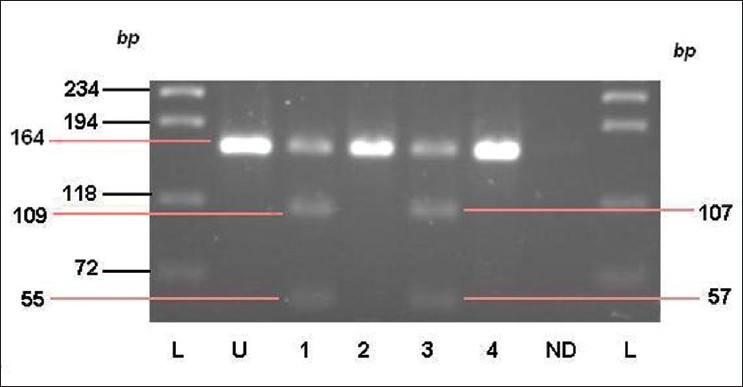
Gel electrophoresis of BfmI and MspI digested PCR products (for representational purpose only) Lane L - ladder; Lane U - undigested PCR product; Lane ND - no-DNA control; Lane 1 - BfmI digested PCR product of a 1138G>A heterozygote; Lane 2 - BfmI digested amplification product of a normal individual; Lane 3 - MspI digested product of a 1138G>C heterozygote; Lane 4 - MspI digested amplification product of a normal individual.

Genomic DNA was isolated from whole blood using standard salting-out method^7^. All 130 DNA samples were tested for the two known mutations (1138G>A and 1138G>C) in the FGFR3 gene by Polymerase Chain Reaction-Restriction Fragment Length Polymorphism (PCR-RFLP) technique as per the previously described protocol.^8^ Results of the analysis for the common mutation of ACH are shown in Figure 1.

## RESULTS

Table 1 shows the number of cases suspected to have ACH and referred for molecular testing since 1998. The numbers increased gradually over the years (mean = 17 patients per year). This could be attributed to increasing awareness among the clinicians and patients in India, about the availability of molecular confirmation of clincial diagnosis of ACH.

**Table 1:** ACH genetic testing carried out in the period 1998–2007 at SGRH

Year	Total (n)	Male (n)	Female (n)
1998	4	2	2
1999	6	0	5
2000	12	5	5
2001	18	5	9
2002	15	2	8
2003	19	8	7
2004	20	8	7
2005	23	5	12
2006	36	14	13
2007	17	5	8
Total tested (n)	130	54	76
Positive, n (%)	53 (40.8)	19 (35.2)	34 (44.7)

Of the 130 individuals, who were clinically suspected to have ACH, molecular confirmatory diagnosis was obtained only in 53 (40.8%) patients. The common mutation 1138G>A was found in 50 (94.3%) of all the positive cases, while three (5.7%) had the rare 1138 G>C substitution mutation.

## DISCUSSION

There are many causes of short-limbed dwarfism. They are commonly differentiated by their clinical and radiological features. However, there is considerable clinical and radiological overlap among the different skeletal dysplasias, and molecular analysis is the only way to confirm the specific diagnosis. The molecular test for achondroplasia can be carried out at any age starting from neonatal period to adulthood. The benefits of molecular testing are many. First, it provides a definitive diagnosis; second, it helps to follow up the patients as per suggested guidelines and provide anticipatory care; third, it helps to provide genetic counseling to the family and prevent the recurrence of the disorder in subsequent pregnancies.

As illustrated in the results, of the 130 postnatal cases referred to us with a clinical diagnosis of ACH, a molecular diagnosis was confirmed in only 40% (n=53) cases. The rest of the cases are likely to have some other type of skeletal dysplasia. If the radiological features are strongly suggestive of ACH and the common mutations that cause ACH are absent, sequencing of the FGFR3 gene is recommended.

ACH is an example of a disorder with great genetic homogeneity, as majority of the cases result from a single mutation, glycine to arginine in the transmembrane domain of the FGFR3 gene. In the present study, 94.3% (50 out of 53) of the total positive cases had this mutation. The glycine to cysteine substitution accounts for a larger proportion in the Indian population, i.e., 5.7% versus 1-3% reported in other populations.^6^,^9^ However, this variance needs to be replicated in larger sample size.

ACH is an autosomal dominant disorder and in familial cases, there is a 50% risk of recurrence if one parent is affected. Even in families where parents are normal and a child with ACH is born, they are very worried about recurrence in subsequent pregnancies (although the risk is small). Therefore, many parents opt for prenatal diagnosis in such cases as well. Antenatal diagnosis of ACH (on ultrasonography) can be suspected on the basis of shortening of long bones. However, this usually appears late after 24–26 weeks of gestation. In India, termination of pregnancy after 20 weeks of gestation is illegal. Therefore, the parents cannot rely on ultrasonography to make a prenatal diagnosis. Molecular genetic testing allows prenatal diagnosis for ACH in high-risk families at about 11 weeks of gestation.

Additional benefits of molecular confirmation of the type of dysplasia are in management and anticipatory care of the patient using specific charts to judge growth of body and head, evaluating for complications, and consideration of therapy with growth hormones (GH), and other drugs.

Most children with ACH have delayed motor milestones, middle-ear dysfunction, and bowing of the lower legs. More rarely, serious health hazards related to hydrocephalus, craniocervical junction compression, upper-airway obstruction, or thoracolumbar kyphosis can occur. Hence, anticipatory care should be directed at identifying children who are at high risk and intervening to prevent serious sequelae.^10^

Many infants with ACH develop a thoracolumbar kyphosis during the first year of life due to unsupported sitting before there is adequate trunk muscle strength. If severe kyphosis appears to be developing, a pediatric orthopedic surgical assessment is recommended to determine if bracing is needed. Severe thoracolumbar kyphosis can give rise to other complications such as spinal stenosis.

In early childhood (< 5 years), some bowing of the legs and instability of the soft tissues surrounding the knee and internal tibial torsion is anticipated. This positional deformity and instability can sometimes leads to difficulty in walking, uncontrolled knee movements, or chronic pain. According to the guidelines outlined by Trotter, Hall and the Committee on Genetics (2005),^10^ pediatric orthopedic consultion is recommended between the age of 1 and 5 years. The child's hips need to be checked for external rotation and hip-flexion contractures. If necessary, a pediatric orthopedist may prescribe exercises that help decrease lumbar lordosis and hip-flexion contractures

An orthopedic evaluation when the child is about 5–13 years of age will help to make appropriate treatment plans if necessary. The common complication of spinal stenosis manifests in adults with numbness, weakness, and altered deep tendon reflexes. If diagnosed at an early stage, complications such as compression of the spinal cord or nerve roots due to lumbosacral spinal stenosis in adults may be treated by surgical decompression.^10^

In children with ACH, growth of the proximal part of the extremities is predominantly compromised in contrast to the only marginally affected growth of the trunk. Hertel *et al.* (2005)^11^ showed that four years of GH treatment in children with ACH improved height without any adverse effect on trunkleg disproportion. It has been reported that GH therapy may result in some increase in growth rate; however, the effects diminish with continued treatment. In Japan, GH therapy in ACH is popular, although in Europe and USA, it is presently not recommended. Limb lengthening surgical procedures such as monofocal and bifocal callotasis, femur, tibial, and humeral lengthening for short stature may be offered after puberty. It can result in substantial increase in height; however, there is a risk of complication, and it is a costly and invasive procedure requiring long periods of hospital stay.^12^,^13^ The patients and the parents have to make an informed choice regarding these therapeutic options. It is concluded that molecular confirmation of skeletal dysplasias should always be sought, as it provides many benefits in disease management.
